# Nitrogen monoxide as dopant for enhanced selectivity of isomeric monoterpenes in drift tube ion mobility spectrometry with ^3^H ionization

**DOI:** 10.1007/s00216-021-03306-7

**Published:** 2021-04-10

**Authors:** Rebecca Brendel, Sascha Rohn, Philipp Weller

**Affiliations:** 1 Institute for Instrumental Analytics and Bioanalytics, Mannheim University of Applied Sciences, Paul-Wittsack-Strasse 10, 68163 Mannheim, Germany; 2 Hamburg School of Food Science, University of Hamburg, Grindelallee 117, 20146 Hamburg, Germany; 3 Department of Food Chemistry and Analysis, Institute of Food Technology and Food Chemistry, Technische Universität Berlin, TIB 4/3-1, Gustav-Meyer-Allee 25, 13355 Berlin, Germany

**Keywords:** Ion mobility spectrometry, Radioactive ionization, Ion formation, Dopant, Terpenes

## Abstract

The ion mobility spectra of the isomeric monoterpenes α-pinene, β-pinene, myrcene, and limonene in drift tube ion mobility spectrometry (IMS) with ^3^H radioactive ionization are highly similar and difficult to distinguish. The aim of this work was to enhance the selectivity of IMS by the addition of nitrogen monoxide (NO) as dopant and to investigate the underlying changes in ion formation responsible for the modified ion signals observed in the ion mobility spectra. Even though ^3^H-based-IMS systems have been used in hyphenation with gas chromatography (GC) for profiling of volatile organic compounds (VOCs), the investigation of ion formation still remains challenging and was exemplified by the investigated monoterpenes. Nonetheless, the formation of monomeric, dimeric, and trimeric ion clusters could be tentatively confirmed by a mass-to-mobility correlation and the highly similar pattern of ion signals in the monomer region was attributed to isomerization mechanisms potentially occurring after proton transfer reactions. The addition of NO as dopant could finally lead to the formation of additional product ions and increased the selectivity of IMS for the investigated monoterpenes as confirmed by principal component analysis (PCA). The discrimination of monoterpenes in the volatile profile is highly relevant in the quality control of hops and was given as the example for application. The results indicate that additional product ions were obtained by the formation of NO^+^ adduct ions, next to hydride abstraction, charge transfer, or fragmentation reactions. This approach can potentially leverage selectivity issues in VOC profiling of complex matrices, such as food matrices or raw materials in combination with chemometric pattern recognition techniques.

**Supplementary Information:**

The online version contains supplementary material available at 10.1007/s00216-021-03306-7.

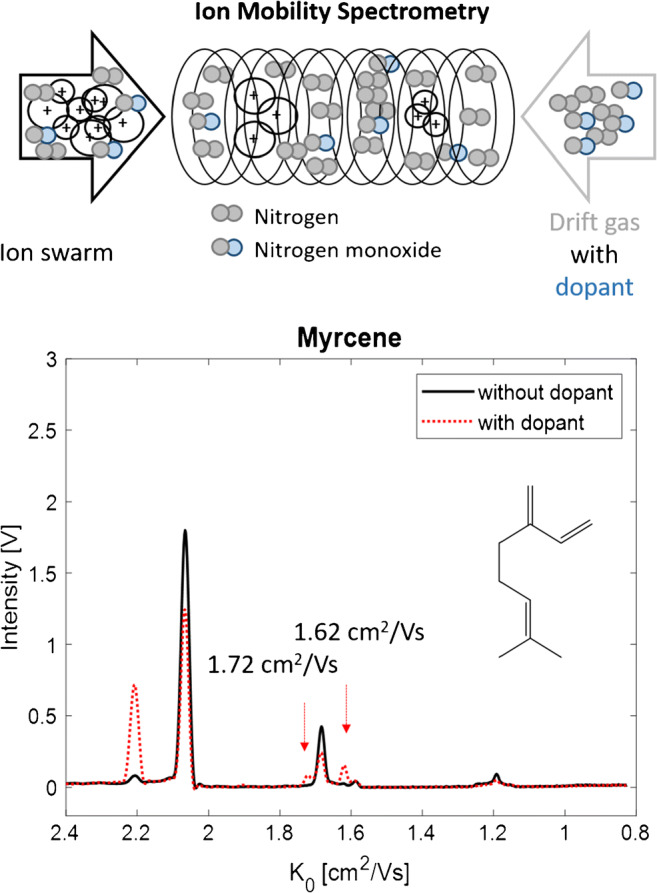

## Introduction

Ion mobility spectrometry (IMS) becomes increasingly important for the analysis of VOCs in a number of application fields. Thereby, monoterpenes are of special interest as flavour or fragrance compounds in several food and cosmetic products [1–3]. Primarily, drift tube IMS operated at atmospheric pressure with radioactive ionization is known as a highly sensitive, selective, and easy-to-handle technique that allows its implementation in portable devices for quality control of food [4]. However, the sensitivity is limited and highly dependent on the proton affinity of the analyte. Commonly, ^63^Ni or the less hazardous ^3^H ionization sources are used as β-particle emitters and are often preferred to non-radioactive ion sources because of the continuous reactant ion production without the requirement of regular maintenance by the operator [5]. By collision of the emitted electrons with the drift or carrier gas molecules, a complex gas phase reaction cascade is initiated and leads to proton-water clusters (H^+^(H_2_O)_n_) as reactant ions in positive mode due to trace levels of water in the surrounding gas atmosphere. The subsequent formation of product ions is given in Eqs. 1 and 2. Analytes entering the ionization region are primarily ionized by proton transfer reactions with the reactant ions to protonated monomer ion clusters (MH^+^(H_2_O)_n-x_). When the analyte concentration further increases, an additional analyte molecule can attach to the monomer, leading to proton-bound dimer ion clusters (M_2_H^+^(H_2_O)_n-x_) [6]. The formation of even higher molecular cluster ions (trimers or tetramers) is rarely observed and is known to be highly structure dependent [7]. With decreasing analyte concentration, the situation is reversed—the number of dimer ions decreases, while the monomer ions increase.

1 $$ M+{H}^{+}{\left({H}_2O\right)}_n\to {MH}^{+}{\left({H}_2O\right)}_{n-x}+x{H}_2O $$

2 $$ M+M{H}^{+}{\left({H}_2O\right)}_n\to {M}_2{H}^{+}{\left({H}_2O\right)}_{n-x}+x{H}_2O $$

After the ionization process, the ions are transferred to the drift tube, where they travel towards the detector under the influence of an electric field at ambient pressure. By collision with the counter-flowing drift gas molecules, they are slowed down depending on their mass, charge, and the structure-related collision cross section (CCS). Thereby, ions are separated and reach the detector with different drift velocities, commonly expressed as reduced mobility *K*_0_ according to Eq. 3 [5, 8]. The degree of hydration of the ion clusters depends on the temperature and moisture in the IMS system. Higher temperatures lead to reduced water concentrations, and therefore to lower degrees of hydration. Furthermore, the number of water molecules is influenced by the structural and chemical properties of the analyte molecule. Drift tube IMS is operated at atmospheric pressure which is why the gas atmosphere in the drift region contains moisture. Consequently, the hydrated ion clusters travel along the drift tube as a hydration-dehydration equilibrium mixture. Because the hydration and dehydration processes occur in the order of 10^−7^ s, only one single peak can be observed in the IMS spectrum for ion clusters of different degrees of hydration [9, 10]. Today, drift tube IMS is routinely coupled to gas chromatography (GC-IMS) as pre-separation to reduce asymmetric ion clustering (ion clusters of different analyte molecules) and to increase selectivity for compounds with highly similar product ions [11].

Although monoterpenes are known as substances with comparatively low proton affinities [7], radioactive ionization-based IMS is a suitable approach for the qualitative and quantitative analysis in highly concentrated samples [1, 2]. In previous studies, we already demonstrated the advantages of headspace GC-IMS with ^3^H ionization (HS-GC-IMS) for the identification of monoterpenes in the volatilome of hop samples for (food) quality control as well as for the quantitation of monoterpene alcohols in cosmetic oils [2, 3]. Furthermore, Rodriguez-Maecker et al. (2017) used HS-GC-IMS for the qualitative analysis of terpenes in essential oils [1]. It is already known that several isomeric monoterpenes feature identical monomer ions in ^3^H-IMS, while they can be differentiated by the number and abundance of their dimer ions [1, 3].

To enhance sensitivity, Vautz et al. (2004) used IMS with photoionization (PI) based on a krypton lamp of 10.6 eV to analyse the monoterpenes α-pinene, β-pinene, 3-carene, and limonene in the ppb_v_ level range in ambient air emitted by wooden surfaces [12]. They observed product ions of nearly identical mobilities for all four investigated monoterpenes. Furthermore, Borsdorf et al. (2005) compared the product ions obtained for several monoterpenes formed by PI and corona discharge (CD) ionization [7]. They could show that CD ionization outperforms PI ionization in the ppt_v_ concentration range by a factor of approx. 300. Furthermore, they observed clearly differing ion signal patterns for all investigated isomers when ionizing with CD. While the ionization pathway of monoterpenes in CD ionization seems to be similar to the fragmentation process observed in electron ionization, in PI primarily [M-H]^+^ monomer ions are formed by hydrogen radical loss [M-H]^+^ and [M(M-H)]^+^ dimers by the attachment of an additional analyte molecule [5, 7].

Another possibility for increasing sensitivity and selectivity in IMS is the usage of dopants without the need for an exchange of the radioactive ionization source. Routinely, low levels of acetone or ammonia are introduced into the ion source to supress substances of low proton affinities, such as aromatic compounds or alcohols [5]. In contrast, the response of low proton affine substances can be enhanced by the addition of nitrogen monoxide (NO), as shown by Gaik et al. (2017) for benzene, toluene, and toluene diisocyanate [13]. NO^+^ reactant ions are formed that can undergo charge transfer reactions, induce hydride abstraction, or form adducts with the analyte leading to alternative product ions.

The aim of this study was to investigate the effects of NO as dopant on the ion formation of isomeric monoterpenes in HS-GC-IMS with ^3^H ionization. Therefore, IMS spectra of the acyclic, monocyclic, and bicyclic monoterpenes α-pinene, β-pinene, myrcene, and limonene occurring in the profile of VOCs of hop were recorded in positive mode with and without the addition of NO and compared to each other. Furthermore, the obtained product ions were compared to values reported in the literature and a mass-to-mobility correlation was done to derive specific information about the chemical composition of the ions. The ion signal pattern of monoterpenes in IMS with radioactive ionization has primarily been used as the characteristic fingerprint in chemometric approaches, but to our knowledge, a detailed investigation of the observed product ions has not been done so far.

## Material and methods

### Reagents and sample preparation

The reference standard mixture of 214 ppm α-pinene (Acros Organics™ by Thermo Fisher GmbH, Kandel, Germany), 230 ppm β-pinene (Alfa Aeser by Thermo Fisher GmbH, Kandel, Germany), 65 ppm myrcene (Sigma Aldrich GmbH, Steinheim, Germany), and 227 ppm limonene (Sigma Aldrich) was prepared in sunflower oil (GLOBUS-Holding GmbH & Co. KG, St. Wendel, Germany) as solvent. Two millilitres was transferred to a 20-mL headspace vial and sealed with a screw cap. The hop sample of the hop cultivar *Citra* was prepared according to Brendel et al. (2020) [3].

### Instrumentation

All measurements were performed on a HS-GC-IMS system, equipped with an OEM ion mobility spectrometer module (Gesellschaft für analytische Sensorysteme mbH, Dortmund, Germany) coupled to a 6890 GC (Agilent Technologies, Waldbronn, Germany) via a heated transfer line (200 °C). HS injection was performed according to Brendel et al. (2020) [3] with a CombiPAL autosampler (CTC Analytics AG, Zwingen, Switzerland). A HP5 column (30 m × 0.32 mm × 0.25 μm) (Agilent Technologies) was installed in the GC oven and nitrogen was used as carrier gas (1 mL min^−1^) with an injection split ratio of 1:50. The oven programme started with an initial temperature of 40 °C followed by a temperature ramp of 10 °C per min to 200 °C (total run time, 16 min). The IMS module was equipped with a tritium ionization source and a drift tube of 9.8 cm length operated at 60 °C, with a drift voltage of 5 kV and a constant drift gas flow of 150 mL min^−1^ nitrogen (N_2_) of 99.9999% gas purity. All spectra were recorded in positive polarity mode with a repetition rate of 47.6 Hz (corresponds to 21 ms per spectrum), an injection pulse width of 150 μs, an injection voltage of 70 arbitrary units (a.u.), and a blocking voltage of 2500 a.u., while six IMS spectra were averaged, respectively. The reduced mobility (*K*_0_) was calculated according to the following equation:

3 $$ {K}_0=\frac{d}{t_d\ast E}\ast \frac{P}{P_0}\ast \frac{T_0}{T} $$

where *d* is the drift tube length (cm), *t*_*d*_ the drift time (s), *E* the electric field strength (V/cm), *P* the pressure (hPa), *P*_0_ the normal pressure (1013.2 hPa), *T*_0_ the normal temperature (273.2 K), and *T* the temperature (K).

Nitrogen monoxide (NO) was added to the drift gas flow as dopant gas (100 ppm in N_2_, Air Liquide Deutschland GmbH, Ludwigshafen, Germany) by a T-screw connection (Swagelok, Solon, OH, USA). Both gas flows were regulated by two separate mass flow controllers (Vögtlin Instruments GmbH, Aesch, Switzerland) and were guided through activated carbon filters (Supelco by Merck KGaA) prior to the entrance of the drift tube. The N_2_ flow was set to 145 mL min^−1^ and the NO flow to 5 mL min^−1^, resulting in a final NO content of approximately 3 ppm in the drift gas flow. A schematic illustration of the setup is shown in Fig. S1 in the Supplementary Information.

### Software

All figures were created by custom toolboxes using MATLAB (The MathWorks Inc., Natick, MA, USA) and Microsoft Office PowerPoint and Excel (Microsoft, Redmond, WA, USA). The Statistics and Machine Learning Toolbox (MathWorks) was used for PCA.

## Results and discussion

### Product ions of isomeric monoterpenes in IMS with ^3^H ionization

**Fig. 1:**
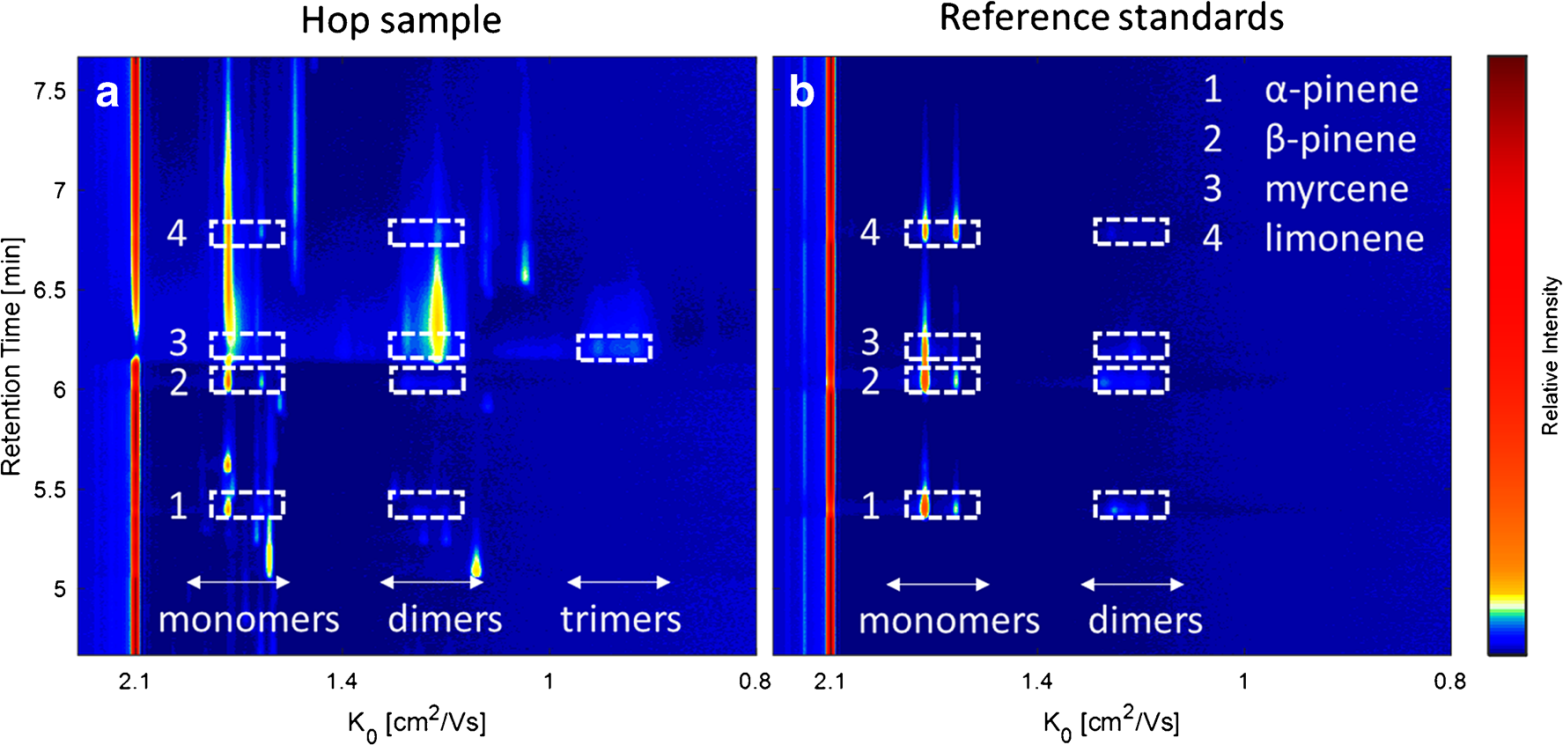
HS-GC-IMS spectrum of the hop sample (**a**) and the reference standards (b) of the monoterpenes α-pinene (1), β-pinene (2), myrcene (3), and limonene (4) (65–230 ppm)

The volatile profile of a pelletised hop sample of the hop cultivar *Citra* was analysed by HS-GC-IMS and the monoterpenes α-pinene, β-pinene, myrcene, and limonene were identified by comparison of the retention times and the ion signal pattern with a reference standard mixture. In Fig. 1, the elution region of the investigated monoterpenes is shown as the topographic view of the two-dimensional GC-IMS spectrum of the hop sample (**a**) and the reference compounds (**b**). The retention time is plotted against the reduced mobility, respectively.

Without distinct knowledge of the actual underlying ion structure, the signals were divided into monomer, dimer, and trimer signals based on the monomer-dimer distribution observable along the retention time axis, which is typical for IMS data with radioactive ionization [2, 12, 14, 15]. At high analyte concentration levels, monomer and dimer signals are typically observed. Trimer ions are only visible at much higher concentrations—usually when the monomer signals have disappeared almost entirely and the dimer signals feature high intensities. With decreasing analyte concentration in the ion source, the dimer signals disappear, while the monomer signals decrease only slowly with a pronounced tailing observable in the spectrum. Consequently, the ion signal pattern in IMS is highly dependent on the analyte concentration level. This should be considered for the comparison of the spectra of the real sample and the reference standards. The obtained ion signals are listed in Table 1 with the reduced mobilities (*K*_0_) and intensities for the monoterpenes in the hop samples and in the reference standard mixture. Only minor deviations (below 1%) were observed between the two compared spectra concerning the retention times and drift times of the investigated monoterpenes. Furthermore, it should be noted that only the most intense peaks were considered with an intensity above 0.04 V that correspond to a signal-to-noise ratio of approximately 7. The general resolving power of the IMS module was R = 76, defined as the ratio of the drift time of the H^+^(H_2_O)_n_ reactant ion peak and the full peak width at half maximum.

**Table 1:** Retention times and reduced mobilities (*K*_0_) of the investigated monoterpenes in the hop sample and the correspondent reference standard

Substance	Retention time (min)	Hop sample K_0_ [cm^2^/Vs] (intensity [V])	Reference compounds K_0_ [cm^2^/Vs] (intensity [V])
α-Pinene	5.38	1.67 (0.53), 1.58 (0.10), 1.22 (0.07), 1.18 (0.05)	1.67 (0.95), 1.58 (0.23), 1.22 (0.17), 1.18 (0.09)
β-Pinene	6.02	1.67 (0.67), 1.58 (0.18), 1.25 (0.08), 1.18 (0.04)	1.67 (0.89), 1.58 (0.25), 1.25 (0.15), 1.18 (0.09)
Myrcene	6.18	1.67 (0.17), 1.18 (0.32), 0.92 (0.12)	1.67 (0.51), 1.18 (0.10), 0.92 (−)
Limonene	6.77	1.67 (0.55), 1.58 (0.14), 1.23 (0.06)	1.67 (0.49), 1.58 (0.47), 1.23 (0.04)

**Fig. 2:**
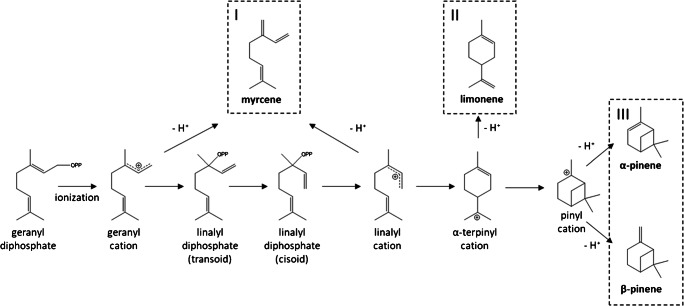
The reaction mechanisms in biosynthesis of acyclic (I), monocyclic (II), and bicyclic (III) monoterpene by the example of myrcene, limonene, α-pinene, and β-pinene according to Degenhardt et al. [18]

Although the molecular structure of the investigated isomers highly differs from each other (see Fig. 2), for the bicyclic α-pinene and β-pinene and monocyclic limonene, identical signals at 1.67 cm^2^/Vs and 1.58 cm^2^/Vs were obtained in the monomer region of the reference standard spectrum. The exception here is the acyclic myrcene, where only one monomer signal at 1.67 cm^2^/Vs could be observed. In the dimer region, several very low intensive signals between 1.25 and 1.18 cm^2^/Vs were observed, differing in number and intensity for the investigated monoterpenes. Consequently, the isomers can only be differentiated by their characteristic dimer ion signal pattern, together with the differing retention times. In the GC-IMS spectrum of the hop sample (Fig. 1A), most monoterpene signals can be observed at their corresponding retention times, while the signal intensities clearly deviate compared to those observed in the reference spectra for α-pinene, β-pinene, and limonene. Especially, the characteristic dimer signals are of very low intensity. In contrast, the intense and strong tailing signals of myrcene due to its high abundance in hops dominate the GC-IMS spectrum with a high GC peak width along the retention time axis (approx. 1.5 min), including the formation of trimeric product ions along the reduced mobility axis. The slightly increased IMS peak widths in the hop sample compared to the reference standard are caused by increased Coulomb repulsion occurring in the larger ion packets at higher ion concentration levels. However, limonene can be identified by its second monomer signal, while the other signals are superimposed by the strong signals of the myrcene product ions. Due to the low proton affinity of monoterpenes in radioactive ionization, there are only a few mobility values reported in the literature. Borsdorf et al. (2002) reported reduced mobility values for α- and β-pinene of 1.69 and 1.59 cm^2^/Vs in a ^63^Ni ionization system which are comparable to the values observed in the current study of 1.67 and 1.58 cm^2^/Vs for the two monomer ions [16]. Furthermore, identical mobilities of product ions of isomeric hydrocarbons were also reported in an earlier work by Borsdorf and Rudolph (2001), who investigated ^63^Ni-IMS spectra of isomeric alkylated benzenes [8]. They observed product ions of similar mobility but of different intensity and concluded that small structural differences of the analyte molecules lead only to small changes in the ion abundance. In a later work, Borsdorf and Neitsch (2009) investigated aliphatic and cyclic isomeric hydrocarbons with different oxygenated substituents, where they obtained nearly identical reduced mobilities, too [15]. They postulated that a complex underlying ion chemistry such as elimination or substitution reactions during ionization leads to the very similar mobility spectra. In this context, it should be noted that monoterpenes are formed via one precursor ion in the biosynthesis, the α-terpinyl cation or the isomeric acyclic linalyl cation, by simple hydride abstraction (Fig. 2). It is conceivable that the precursor ion is formed by proton transfer reactions followed by rearrangement during the ionization process leading to the identic product ions for isomeric monoterpenes in IMS. This assumption is in accordance with the results described by Müller et al. (2009) who compared the fragmentation patterns of the protonated monomer ions of α-pinene, β-pinene, myrcene, and limonene after collision-induced dissociation (CID) in tandem MS [17]. They obtained similar fragment ions with highly similar relative ratios that indicate identical ion structures for the monomer ions of all four analytes most probably caused by isomerization occurring after the proton transfer reaction with the H^+^(H_2_O)_n_ reactant ions.

**Fig. 3:**
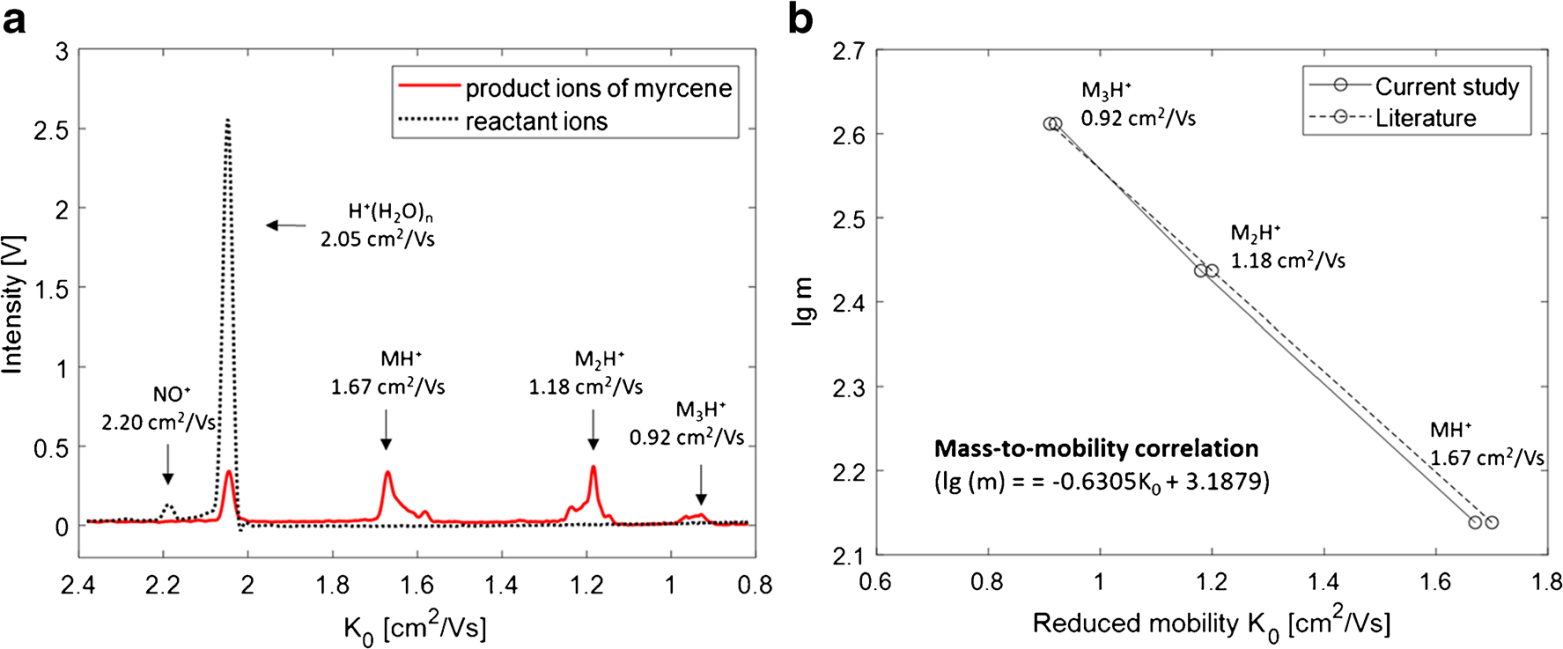
The IMS spectrum of myrcene in the hop sample (**a**) as red line with the initial reactant ions as black dotted line and the corresponding mass-to-mobility correlation (**b**) obtained in the current study (solid line) and in the literature (dotted line) according to Borsdorf et al. [7]

To investigate the product ion composition in this study, the corresponding mass spectrum could be helpful. However, there would be no further information about the chemical structure of the ion. Additionally, the transfer of ions from atmospheric pressure to the high vacuum of the mass spectrometer (MS) poses a challenge. Weakly bound cluster ions may decompose and the adiabatic cooling may lead to ion-neutral association in the transfer region [7]. Therefore, a reliable assignment of masses to the ion signals in drift tube IMS still remains difficult. However, Borsdorf et al. (2005) used a PI-IMS-MS hyphenation system to investigate the ion formation of monoterpenes in IMS with PI [7]. They report reduced mobility values of 1.70 and 1.17 cm^2^/Vs for limonene, 1.70 and 1.20 cm^2^/Vs for α-pinene, and 1.67, 1.69, 1.19, and 0.91 cm^2^/Vs for β-pinene with dominating *m/z* ratios of 135 ([M-H]^+^), 271 ([M(M-H)]^+^), 272 (M_2_^+^), and 273 ([M_2_H]^+^). The dominating monomeric and dimeric signals in the mass spectra were accompanied by a series of less intensive signals of higher masses. Furthermore, they derived an excellent mass-to-mobility correlation (lg(m) = −0.6053 *K*_0_ + 3.1625) between the identified ionic masses and the corresponding reduced mobility values of the pinene isomers. The mass-to-mobility correlation is a common method in IMS to describe the relationship between ion mass and mobility. Vautz et al. (2004) also used PI-IMS for the analysis of monoterpenes and report reduced mobilities of 1.66, 1.20, and 0.92 cm^2^/Vs for α- and β-pinene, and 1.66, 1.16, and 0.92 cm^2^/Vs for limonene [12]. The values are very close, with slight deviations to those reported by Borsdorf et al. (2005) as well as to those obtained in the current study [7]. According to this observation, the ion masses of monomers (MH^+^), dimers (M_2_H^+^), and trimers (M_3_H^+^) generally assumed to be formed in ^3^H-IMS were correlated to the observed reduced mobility values of myrcene in the GC-IMS spectrum of the hop sample. The resulting mass-to-mobility correlation and the mobility spectrum of myrcene are plotted in Fig. 3.

The obtained regression equation (lg(m) = −0.6305 *K*_0_ + 3.1879) is very close to that reported in the literature by Borsdorf et al. (2005) [7]. Consequently, this correlation confirms the assumption that the observed ion signals in the IMS spectrum of myrcene and its isomers correspond to the monomeric, dimeric, and trimeric product ions. Additionally, it can be assumed that the ions occurring close to the dominating product ions are formed by reaction mechanisms occurring next to proton transfer reactions as also observed in the PI-IMS-MS spectra of terpenes [7]. Possibly, different configurations of the ion cluster that highly depend on the analyte molecule structure can lead to differing CCS and finally to differing mobilities of the dimer ion clusters. Theoretical modelling of energetically stable gas phase cluster configurations and the calculation of corresponding CCS could provide further information and are part of future research.

In addition to the mobility spectrum of myrcene, the initial reactant ion spectrum is plotted as a black dotted line in Fig. 3a. Next to the dominating H^+^(H_2_O)_n_ reactant ion peak (RIP) at 2.05 cm^2^/Vs, a small peak at 2.20 cm^2^/Vs can be observed. According to Borsdorf et al. (2006) and Gaik et al. (2017), this can be attributed to NO^+^ formed, resulting from trace levels of NO in the surrounding gas atmosphere of the ionization source [5, 13]. However, this signal disappeared in the mobility spectrum of myrcene (red line). There are two possible explanations: First, NO^+^ ions could not have been formed due to a competitive ionization situation between NO and the highly concentrated analyte in the ionization region. Secondly, NO^+^ has entirely been consumed by reactions with the analyte molecule. It is known from the literature that primarily low proton affine substances undergo reactions with NO^+^ that are not easily ionized by proton transfer reactions [13]. To identify product ions in the mobility spectrum of monoterpenes that occur exclusively due to interactions with NO^+^ reactant ions, NO was additionally introduced as dopant in the nitrogen drift gas flow.

### Product ions of isomeric monoterpenes in GC-IMS with NO as dopant gas

**Fig. 4:**
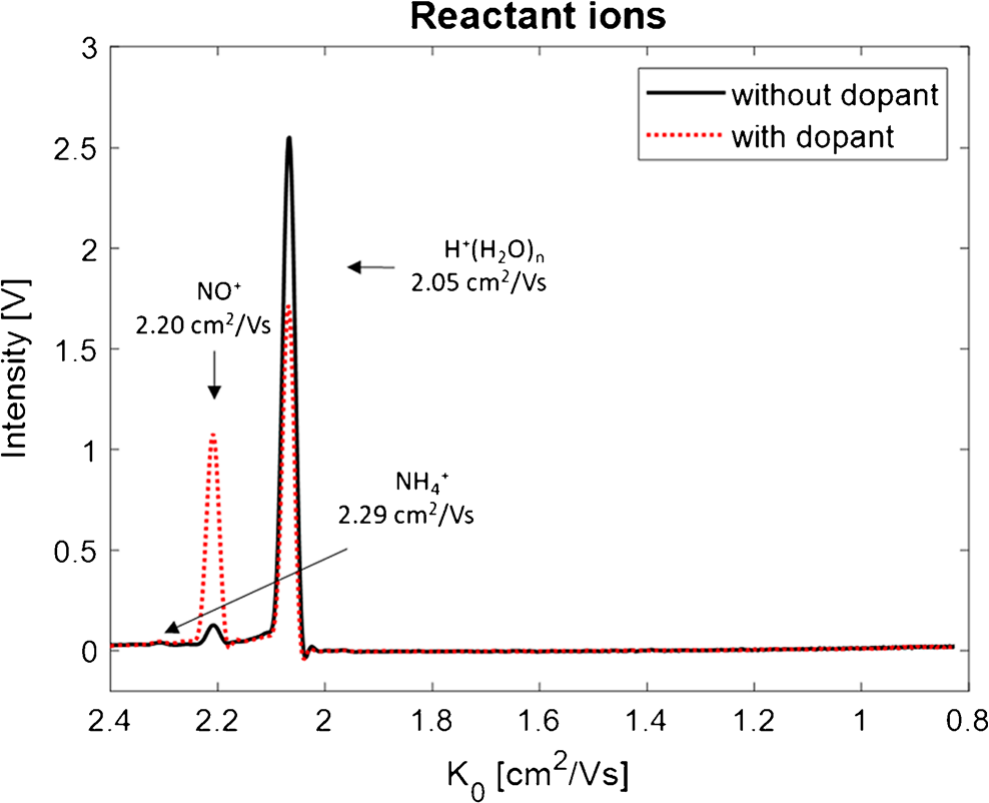
Reactant ion spectrum with 3 ppm NO dopant in N_2_ drift gas

An additional gas flow of 5 mL min^−1^ of a 100 ppm dopant gas NO in N_2_ was introduced into the drift tube of the GC-IMS setup leading to a concentration of approximately 3 ppm NO in the resulting drift gas stream. The NO^+^ RIP at 2.20 cm^2^/Vs increased clearly as illustrated in Fig. 4 (red dotted line), while the H^+^(H_2_O)_n_ RIP at 2.05 cm^2^/Vs decreased (black line). Consequently, NO^+^ reactant ions were formed by diffusion of NO from the drift tube into the surrounding gas atmosphere of the ionization region.

**Fig. 5:**
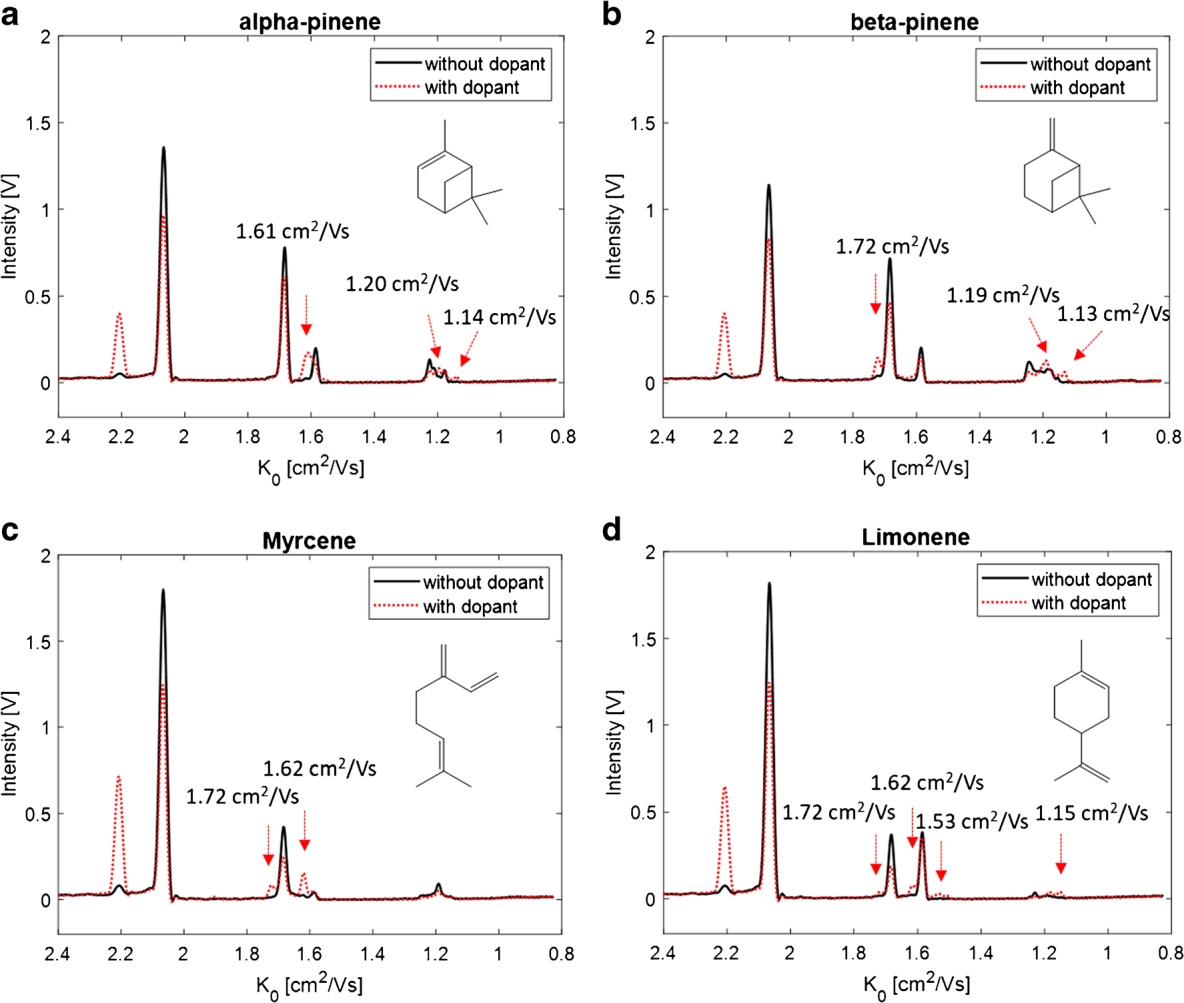
IMS spectra of α-pinene (**a**), and β-pinene (**b**), myrcene (**c**), and limonene (**d**) with 3 ppm NO as dopant in the drift gas (red dotted line) and without the dopant (black line)

The hop sample, as well as the reference standard mixture of monoterpenes, was measured again with GC-IMS, after stabilization of the NO^+^ RIP at an intensity of 1.08 V. The IMS spectra obtained in the standard mixture with (red dotted line) and without (black line) the dopant NO are plotted for the investigated monoterpenes in Fig. 5.

**Fig. 6:**
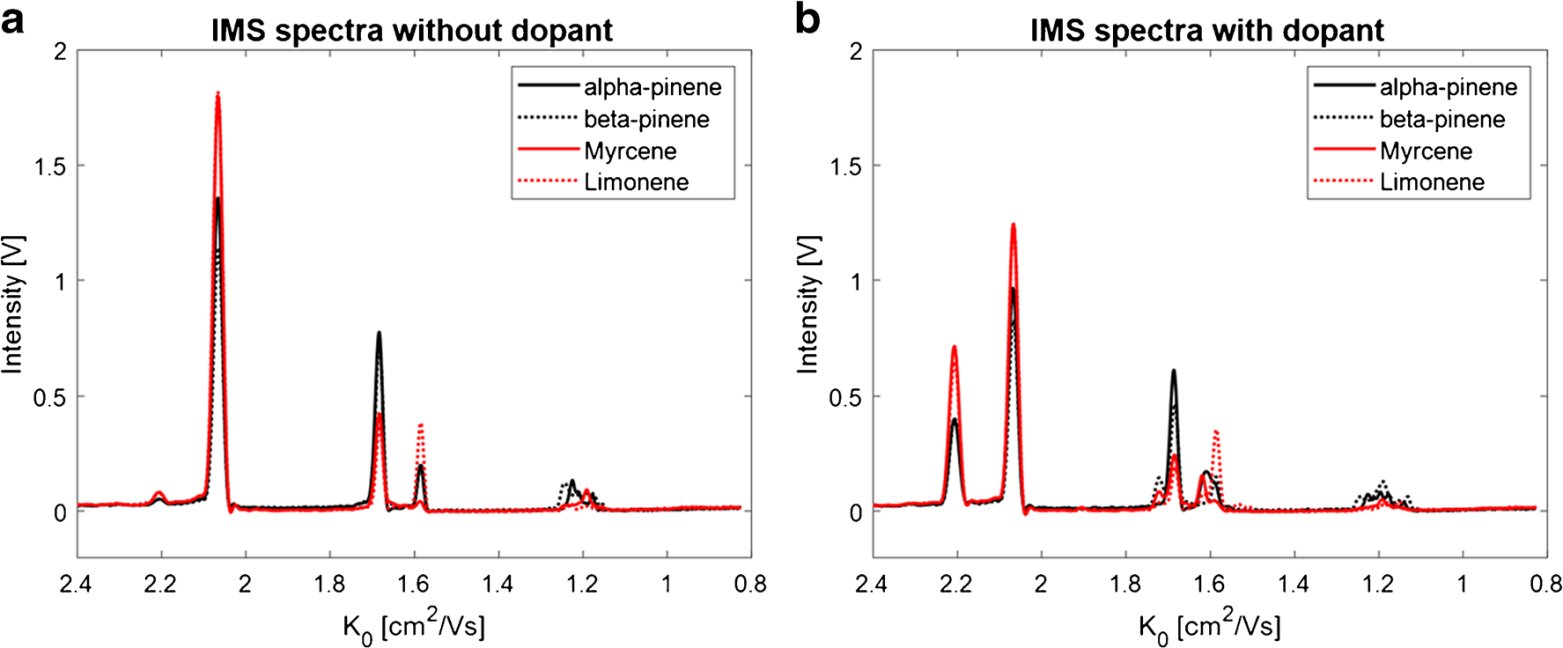
Superimposed IMS spectra of all investigated monoterpenes α-pinene (black solid line), and β-pinene (black dotted line), myrcene (red solid line), and limonene (red dotted line) without dopant (**a**) and with dopant (**b**)

The addition of NO did not lead to a general signal increase. Instead, it caused the formation of additional signals that are marked with arrows in Fig. 5a–d. The formation of the additional signals was accompanied by a decrease of the NO^+^ RIP indicating the consumption of NO^+^ ions. Furthermore, the drift times of the H^+^(H_2_O)_n_ ions, as well as of the product ions of H^+^(H_2_O)_n_, did not change after the addition of NO to the drift gas. Most probably trace levels of NO have no significant impact on the general drift times obtained with nitrogen. In the IMS spectrum of α-pinene (**a**), additional signals at 1.61, 1.20, and 1.14 cm^2^/Vs were formed, in the spectrum of β-pinene (**b**) at 1.72, 1.19, and 1.13 cm^2^/Vs, in the spectrum of myrcene (**c**) at 1.72 and 1.62 cm^2^/Vs, and in the spectrum of limonene (**d**) at 1.72, 1.62, 1.53, and 1.15 cm^2^/Vs. It can be assumed that these signals were already formed before the addition of the dopant. Most probably, they were formed in such few amounts that they were not detectable. However, these additional signals allow for the clear differentiation of all four monoterpenes, especially in the monomer region between 1.72 and 1.53 cm^2^/Vs as illustrated in the superimposed spectra plot in Fig. 6b, being not possible in the original approach without the dopant gas (Fig. 6a).

The product ions formed with NO^+^ seem to be more structure-dependent. Again, the question concerning the product ion composition arises: From the literature, it is known that NO^+^ can undergo charge transfer reactions with the analyte M leading to M^+^ product ions. Furthermore, hydride abstraction can be induced leading to [M-H]^+^ product ions or adduct ions of the form [MNO]^+^ can be formed [13]. Consequently, it can be assumed that all signals in the monomer region with higher reduced mobility values than that of the suspected MH^+^ ion at 1.67 cm^2^/Vs can be assigned to product ions of lower masses, such as [M-H]^+^ or M^+^ ions. However, it remains questionable whether the resolution of the IMS is sufficient to distinguish between MH^+^, [M-H]^+^, or M^+^ ions. It is more likely that those signals belong to fragment ions of lower masses, as reported by Karasek et al. (1974) for reaction products for alkanes in ^63^Ni-IMS [19]. They assumed that hydride abstraction reactions are sufficiently exothermic to cause fragmentation. Furthermore, signals with lower reduced mobility values than 1.67 cm^2^/Vs can be assigned to product ions of higher masses, such as the adduct ion [MNO]^+^. Ion-neutral interaction of analyte ions and NO in the drift tube can be excluded because this would lead to a continuum in IMS and no resolved signals could be observed.

**Fig. 7:**
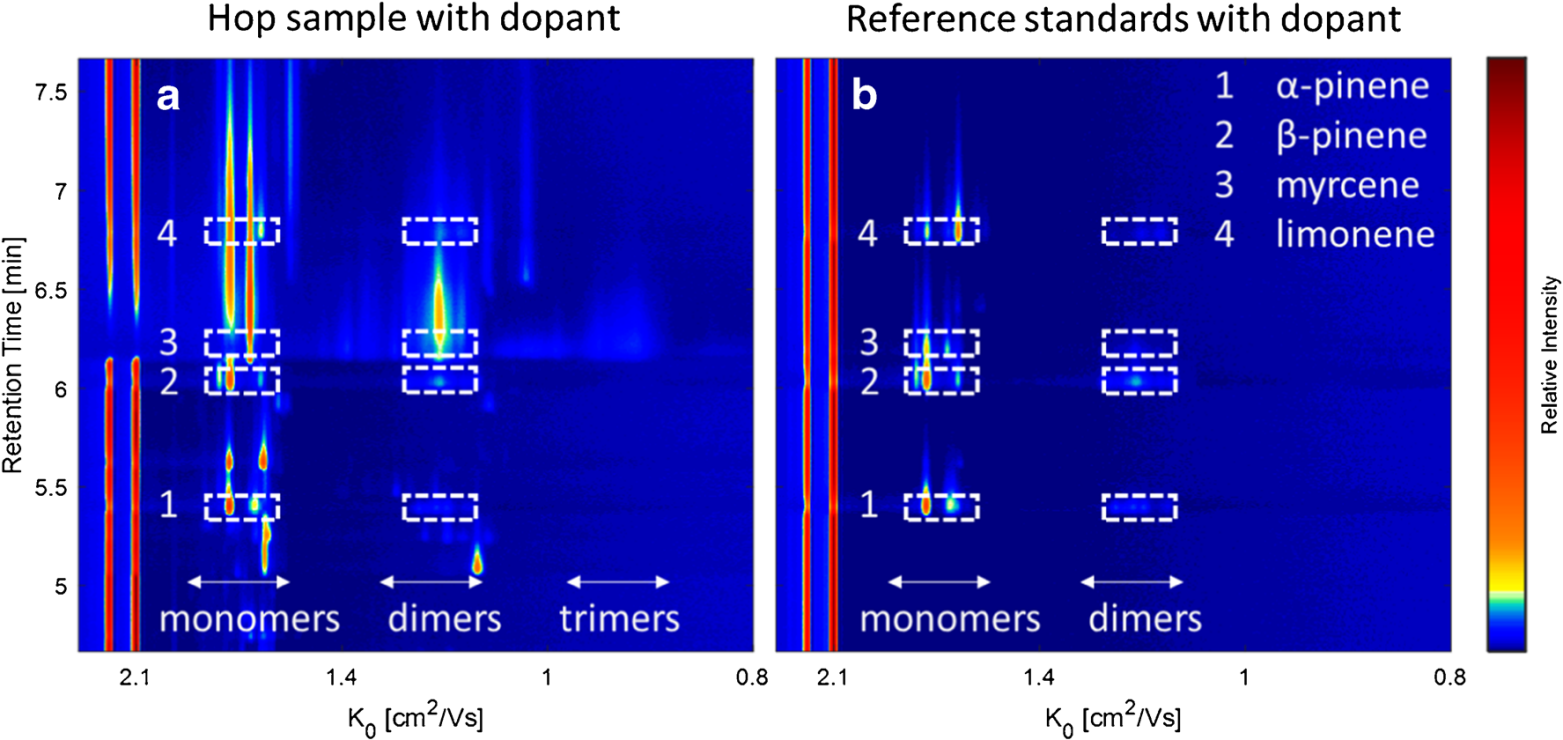
HS-GC-IMS spectrum of the hop sample (**a**) and the reference standards (b) of the monoterpenes α-pinene (1), β-pinene (2), myrcene (3), and limonene (4) (65–230 ppm) with 3 ppm NO dopant in the drift gas

Although the exact chemical structure of the ion(s) is unknown at the moment, usage of NO as dopant increases the selectivity of IMS for isomeric monoterpenes. To underline the benefits of this observation, the GC-IMS spectra of a selected (monoterpene-rich) hop sample and the reference standard mixture under the influence of the NO dopant are shown in Fig. 7a and b. Due to the additional signals primarily in the monomer region, α-pinene, β-pinene, and myrcene can be clearly identified and differentiated compared to the corresponding spectra without dopant (Fig. 1), while there was no improvement for limonene due to the strong overlap with the signals of myrcene. However, it can still be identified by its monomer signal at 1.58 cm^2^/Vs.

**Fig. 8:**
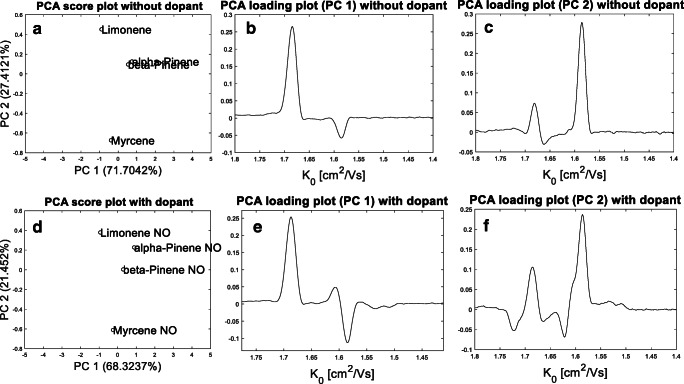
Score plot of the first and second principal components (PC) (a) with corresponding loading plots (**b**, **c**) of the monomer region of the reference spectra of α-pinene, β-pinene, myrcene, and limonene without NO and score plot of the first and second principal components (PC) (**d**) with corresponding loading plots (e, f) of the monomer region of the reference spectra of α-pinene, β-pinene, myrcene, and limonene with NO

It should be noted that the investigated monoterpenes could all be differentiated along the retention time dimension of the GC-IMS spectrum. The used GC-IMS device was based on a temperature-ramped gas chromatograph equipped with capillary columns of small inner diameter providing high chromatographic resolution. However, due to its simple operation principles, drift tube IMS is often used in standalone, handheld IMS devices, or portable GC-IMS devices equipped with capillary columns of higher inner diameter operated in isothermal mode. In a previous study, where we compared the temperature-ramped GC-IMS to a portable GC-IMS device, we could already demonstrate that the portable device provides clearly less chromatographic resolution [20]. Consequently, standalone devices or devices of low chromatographic resolution will benefit from the enhanced selectivity in IMS after the addition of NO as dopant, in particular, in combination with chemometric data analysis, which is often based on pattern recognition. For illustration, PCA was applied to the monomer region of the IMS reference spectra of the investigated monoterpenes with and without the usage of NO. In this particular case, IMS spectra (K_0_ x V) were used at the peak centre without the chromatographic information to allow a discrimination according to the ion drift times only. The obtained score plots and the corresponding loading plots are given in Fig. 8.

Without NO, the IMS spectra were not clearly separable by PCA (Fig. 8a), mainly due to highly similar score values for α-pinene and β-pinene in the first two principal components (PC). The loading plot of PC 1 (Fig. 8b) indicates that the monomer signal at 1.67 cm^2^/Vs had the highest impact on the discrimination in the first PC. The loading plot of PC 2 (Fig. 8c) indicates that the monomer signal at 1.58 cm^2^/Vs had the highest discriminative power in the second PC. In contrast, all IMS spectra were clearly separable in the PCA score plot, when NO was used as dopant (Fig. 8d). The corresponding loading plots (Fig. 8e and f) indicate that the additional signals at 1.61, 1.62, and 1.72 cm^2^/Vs are responsible for the discrimination in the first and second PC.

## Conclusion

Based on mass-to-mobility correlations and the comparison with values from literature, the dominating product ions obtained for isomeric monoterpenes in GC-IMS with ^3^H radioactive ionization could be assigned to monomeric, dimeric, and trimeric product ions. Although the investigated monoterpenes clearly differ in their molecular structure, they form product ions of highly similar reduced mobilities. They can only be differentiated by their dimeric ion signal pattern next to their deviating retention times. Unfortunately, the ion formation in IMS is highly dependent on the analyte concentration and leads to less intensive dimer ion signals at low concentration levels. It could be demonstrated by PCA that the use of NO as dopant clearly increases selectivity in GC-IMS for the investigated isomeric monoterpenes, due to the formation of additional characteristic, structure-related product ions. Furthermore, the relevance of this observation was demonstrated for the volatile profile of hop, where the dimeric ions were only less abundant due to the low concentration. The addition of NO led to the formation of additional product ions in the more abundant monomer region, allowing substance identification even at low concentration levels. The observed ions were tentatively assigned to product ions formed by interactions of the analyte molecule and NO^+^ in the ionization source, such as adduct ions, next to reaction products generated by hydride abstraction, charge transfer reactions, or fragmentation. Finally, this study could demonstrate in a first step the benefits of using NO as dopant in IMS for increasing selectivity for monoterpenes, in particular, when chemometric measurements are performed in complex samples. In view of the growing demand of drift tube IMS as simple and cost-effective detection technique for VOCs in portable devices in quality control of food, further research is required to unequivocally elucidate the product ion structures.

## Supplementary information

ESM 1 (PDF 92 kb).

## References

[1] Rodríguez-Maecker R, Vyhmeister E, Meisen S, Rosales Martinez A, Kuklya A, Telgheder U (2017). Identification of terpenes and essential oils by means of static headspace gas chromatography-ion mobility spectrometry. Anal Bioanal Chem.

[2] Brendel R, Schwolow S, Rohn S, Weller P (2020). Comparison of PLSR, MCR-ALS and kernel-PLSR for the quantification of allergenic fragrance compounds in complex cosmetic products based on nonlinear 2D GC-IMS data. Chemom Intell Lab Syst.

[3] Brendel R, Schwolow S, Rohn S, Weller P (2020). Gas-phase volatilomic approaches for quality control of brewing hops based on simultaneous GC-MS-IMS and machine learning. Anal Bioanal Chem.

[4] Wang S, Chen H, Sun B. Recent progress in food flavor analysis using gas chromatography-ion mobility spectrometry (GC-IMS). Food Chem. 2020;315:126158. 10.1016/j.foodchem.2019.126158 32014672

[5] Borsdorf H, Eiceman GA (2006). Ion mobility spectrometry: principles and applications. Appl Spectrosc Rev.

[6] Eiceman GA, Karpas Z, Hill HH (2016). Ion mobility spectrometry, third edition, first issued in paperback.

[7] Borsdorf H, Stone JA, Eiceman GA (2005). Gas phase studies on terpenes by ion mobility spectrometry using different atmospheric pressure chemical ionization techniques. Int J Mass Spectrom.

[8] Borsdorf H, Rudolph M (2001). Gas-phase ion mobility studies of constitutional isomeric hydrocarbons using different ionization techniques. Int J Mass Spectrom.

[9] Valadbeigi Y, Ilbeigi V, Michalczuk B, Sabo M, Matejcik S (2019). Effect of basicity and structure on the hydration of protonated molecules, proton-bound dimer and cluster formation: an ion mobility-time of flight mass spectrometry and theoretical study. J Am Soc Mass Spectrom.

[10] Carroll DI, Dzidic I, Stillwell RN, Horning EC (1975). Identification of positive reactant ions observed for nitrogen carrier gas in plasma chromatograph mobility studies. Anal Chem.

[11] Vautz W, Franzke J, Zampolli S, Elmi I, Liedtke S. On the potential of ion mobility spectrometry coupled to GC pre-separation - a tutorial. Anal Chim Acta. 2018;1024:52–64. 10.1016/j.aca.2018.02.052 29776547

[12] Vautz W, Sielemann S, Baumbach JI (2004). Determination of terpenes in humid ambient air using ultraviolet ion mobility spectrometry. Anal Chim Acta.

[13] Gaik U, Sillanpää M, Witkiewicz Z, Puton J (2017). Nitrogen oxides as dopants for the detection of aromatic compounds with ion mobility spectrometry. Anal Bioanal Chem.

[14] Pomareda V, Guamán AV, Mohammadnejad M, Calvo D, Pardo A, Marco S. Multivariate curve resolution of nonlinear ion mobility spectra followed by multivariate nonlinear calibration for quantitative prediction. Chemom Intell Lab Syst. 2012;118:219–29.

[15] Borsdorf H, Neitsch K (2009). Ion mobility spectra of cyclic and aliphatic hydrocarbons with different substituents. Int J Ion Mobil Spectrom.

[16] Borsdorf H, Nazarov EG, Eiceman GA (2002). Atmospheric pressure chemical ionization studies of non-polar isomeric hydrocarbons using ion mobility spectrometry and mass spectrometry with different ionization techniques. J Am Soc Mass Spectrom.

[17] Müller M, Mielke LH, Breitenlechner M, McLuckey SA, Shepson PB, Wisthaler A, Hansel A. MS/MS studies for the selective detection of isomeric biogenic VOCs using a Townsend discharge triple quadrupole tandem MS and a PTR-linear ion trap MS. Atmos Meas Tech. 2009;2:703–712.

[18] Degenhardt J, Köllner TG, Gershenzon J (2009). Monoterpene and sesquiterpene synthases and the origin of terpene skeletal diversity in plants. Phytochemistry..

[19] Karasek FW, Denney DW, DeDecker EH (1974). Plasma chromatography of normal alkanes and its relation to chemical ionization mass spectrometry. Anal Chem.

[20] Gerhardt N, Birkenmeier M, Sanders D, Rohn S, Weller P (2017). Resolution-optimized headspace gas chromatography-ion mobility spectrometry (HS-GC-IMS) for non-targeted olive oil profiling. Anal Bioanal Chem.

