# Supplementary material for: Nitrogen monoxide as dopant for enhanced selectivity of isomeric monoterpenes in drift tube ion mobility spectrometry with 3H ionization
Source: Anal Bioanal Chem. 2021 Apr 10;413(13):3551–60. doi: 10.1007/s00216-021-03306-7 (PMC8105222; doi:10.1007/s00216-021-03306-7)
Supplement: Supplementary file 1 — (PDF 92 kb). [file 216_2021_3306_MOESM1_ESM.pdf]

Supplementary Information

Nitrogen monoxide as dopant for enhanced selectivity of isomeric monoterpenes in drift tube ion mobility spectrometry with ^{3}H ionization

Rebecca Brendel, Sascha Rohn, Philipp Weller

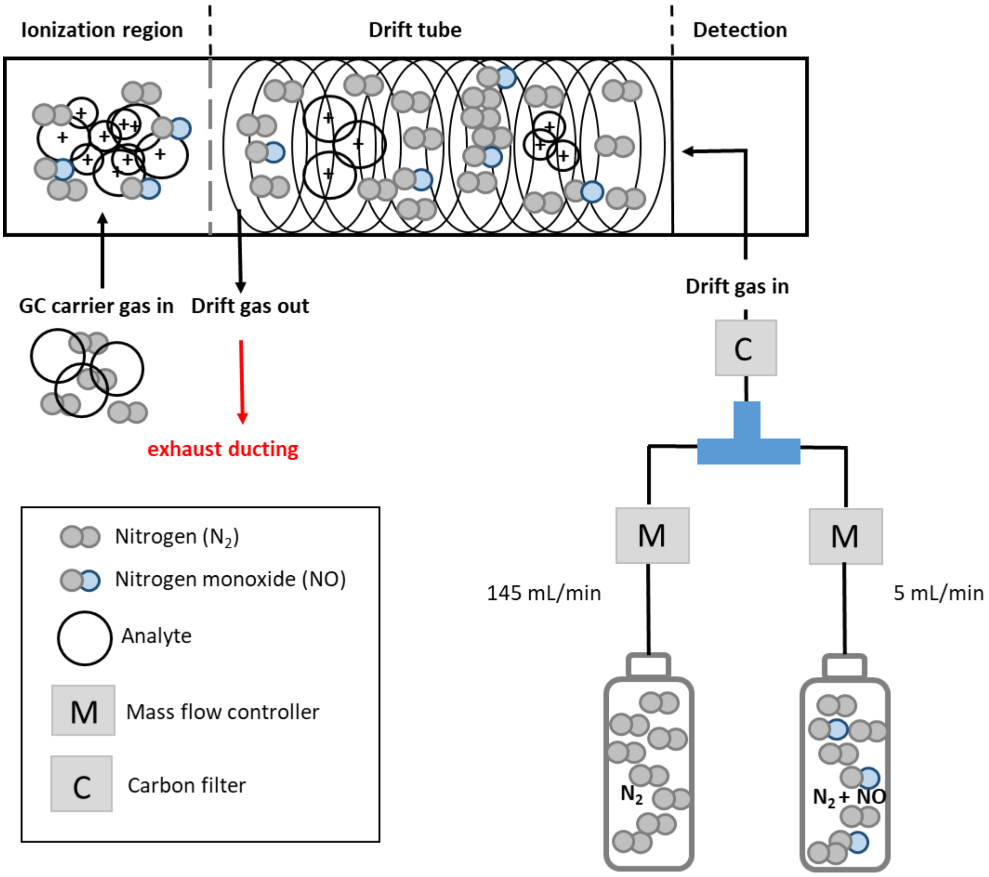

Figure S1 Schematic illustration of the instrument setup.
